# Characteristics of Physician Outflow from Disaster Areas following the Great East Japan Earthquake

**DOI:** 10.1371/journal.pone.0169220

**Published:** 2017-01-03

**Authors:** Saori Kashima, Kazuo Inoue, Masatoshi Matsumoto

**Affiliations:** 1 Department of Public Health and Health Policy, Institute of Biomedical and Health Sciences, Hiroshima University, Hiroshima, Japan; 2 Department of Community Medicine, Chiba Medical Center, Teikyo University School of Medicine, Chiba, Japan; 3 Department of Community Based Medical System, Institute of Biomedical and Health Sciences, Hiroshima University, Japan; Hamamatsu Ika Daigaku, JAPAN

## Abstract

**Objective:**

The shortage of physicians after a major disaster is a crucial issue. We aimed to evaluate the characteristics of physicians who left affected areas following the accident at Fukushima Daiichi Nuclear Power Plant caused by the Great East Japan Earthquake on March 11, 2011.

**Methods:**

Using data from a physician census conducted in 2010 (pre-disaster) and 2012 (post-disaster), we evaluated changes in the number of physicians in affected areas. We then calculated the odds ratios and 95% confidence intervals using a logistic regression model to evaluate the association between physician characteristics and outflow. We also conducted stratified analyses based on physician characteristics.

**Results:**

The number of physicians decreased in Fukushima Prefecture (–5.3%) and increased in Miyagi Prefecture (2.8%). The decrease in Fukushima and increase in Miyagi were evident even after taking the prefecture’s population change into account (change in physician to population ratios: –1.9% and 3.2%, respectively). Compared with physicians who lived in areas >100 km from the nuclear power plant, physicians living 20–50 km and 50–100 km were, respectively, 3.9 times (95% confidence interval, 2.6–5.7) and 2.6 times (95% confidence interval, 1.7–3.8) more likely to migrate to distant areas. In the stratified analysis, younger physicians and those earlier in their careers had higher odds ratios for outflow than other physicians (*P* for interaction = 0.02 and <0.01, respectively).

**Conclusions:**

The risk of outflow was greater among younger and early-career physicians in areas around the power plant. Political support may be necessary to recruit and retain such physicians, who will be responsible for future community health in the disaster area.

## Introduction

**Fig 1:**
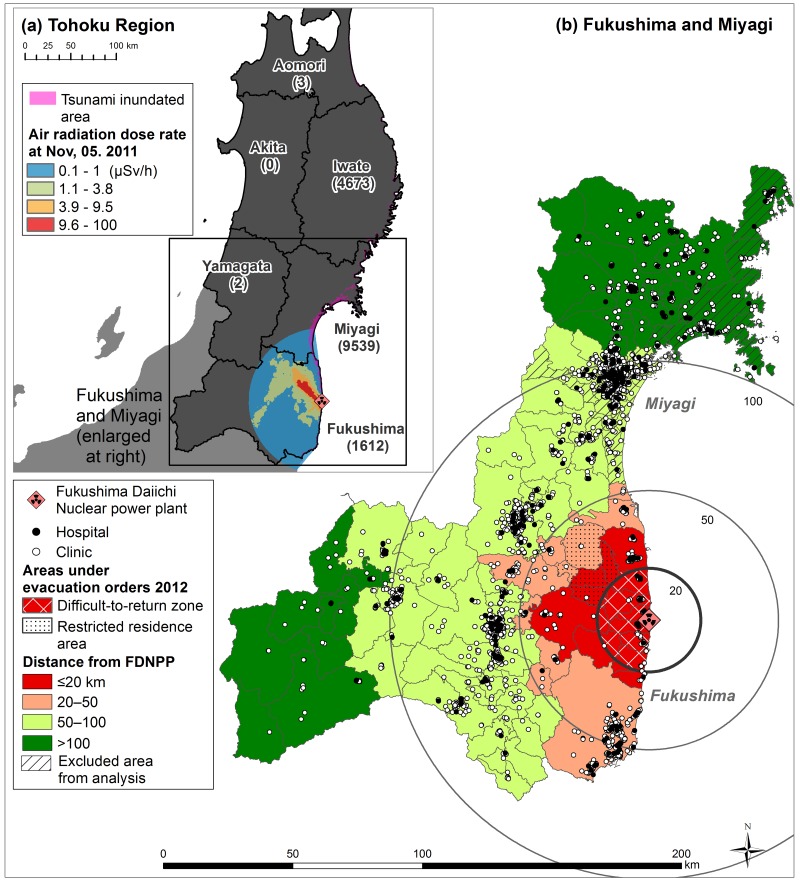
Study area for analysis around Fukushima Daiichi Nuclear Power Plant. (a) Tohoku region; (b) Fukushima and Miyagi prefectures. The number in parentheses in the upper-left part of the figure is the number of people reported killed. Information about tsunami-inundated areas was obtained from the Geospatial Information Authority of Japan and Ministry of Land, Infrastructure, Transport and Tourism; the air radiation dose rate was acquired from the Japan Atomic Energy Agency.

The large-scale Great East Japan Earthquake struck the northeastern part of the country on March 11, 2011. This earthquake caused a massive tsunami, which hit the Pacific coast and damaged Fukushima Daiichi Nuclear Power Plant (FDNPP) [1]. A 15-meter tsunami disabled the cooling system of three Fukushima Daiichi reactors. This resulted in the melting of their cores and a radiation leak; radioactive elements were released northwest and south of the plant (Fig 1) [2, 3]. The accident was rated as the 7th (maximum) caution level on the International Nuclear Events Scale owing to the high level of radioactive substances released on days 4 to 6 [4].

At the time of the disaster, there were no fatalities directly caused by radiation or cases of radiation sickness as a result of the accident. However, about 116,000 people who lived within a 20-km radius of FDNPP, which was officially designated a restricted area, were forced to evacuate their homes (Fig 1). In addition, many other individuals, including health-care professionals, who lived outside the restricted area left their homes owing to fear of the potential radioactive hazard. Providing medical care services is a basic social necessity, and the lack of such care following the nuclear accident has become a serious issue for both local governments and residents [5, 6].

It was unclear what types of physicians were most affected by the FDNPP accident and how they were distributed. There is a need for a political focus on such physicians so as to provide an efficient means of preventing them from leaving following a major accident. Thus, the purpose of this study was to evaluate the association between physicians’ characteristics and their outflow to distant following the nuclear accident.

## Materials and Methods

### Study area

The Great East Japan Earthquake struck the Tohoku region of Japan, which consists of six prefectures (Fukushima, Miyagi, Iwate, Aomori, Akita, and Yamagata; Fig 1). Within this area, the number killed as a result of the earthquake was 15,829 (99.6% of the total killed) and the number of missing people was 2,581 (99.9% of the total missing) [7]. The victims died mainly as a result of the tsunami, which attacked the Pacific coast in this region. FDNPP is located on the Pacific coast of Fukushima Prefecture, and radioactive material was subsequently scattered around that area. To evaluate the influence of the FDNPP accident, we focused on Fukushima and Miyagi prefectures, and we compared the results there with those in Iwate Prefecture and the western Tohoku region (Aomori, Akita, and Yamagata prefectures) as a reference area (Fig 1). Administrative boundaries of municipalities were obtained from the Ministry of Land, Infrastructure, Transport, and Tourism.

### Physician data

We obtained data about physicians from the Survey of Physicians, Dentists and Pharmacists, compiled by the Ministry of Health, Labour and Welfare [8, 9]. The survey is a complete census of physicians conducted every 2 years by the ministry. All licensed physicians are obliged to register for this census. We obtained permission from the ministry to use the census data for research purposes. The Great East Japan Earthquake occurred in March 2011, and so we used data from the physician census in 2010 (pre-disaster) and 2012 (post-disaster). Physicians registered as practicing in hospitals (including university hospitals and as hospital physicians) or clinics (clinic physicians) were recognized as practicing physicians in this study.

### Distance from disaster site

We measured straight distances from the physicians’ workplaces to the FDNPP site (FDNPP distance); we classified the FDNPP distance based on whether the whole or any part of the municipality where the physician worked was included within the specified distance range. For example, if a municipality included an area that was 18 km from FDNPP, the municipality was assigned in the category of ≤20 km in terms of FDNPP distance. We obtained administrative boundaries of municipalities for 2011 from the Ministry of Land, Infrastructure, Transport and Tourism. We measured distances using ArcGIS version 10.1 (ESRI Japan Inc., Tokyo, Japan). To determine the situation in affected areas, we obtained geographic information about tsunami-inundated regions from the Geospatial Information Authority of Japan and Ministry of Land, Infrastructure, Transport and Tourism [10]; we procured the air radiation dose rate on November 5, 2011 from the Japan Atomic Energy Agency [11].

We classified the FDNPP distance into four categories: ≤20 km, 20–50 km, 50–100 km, and >100 km. On April 22, 2011, the government defined the following zones: restricted area with prohibited entry (area within a 20-km radius of FDNPP); evacuation areas; and areas to prepare for evacuation in case of emergency [12] (Fig 1). At the time of the physician census in December 2012, the area within 20 km remained a restricted area. We thus adopted a cutoff point of 20 km for the group of physicians closest to the FDNPP. The zone of 20–50 km included areas with an air radiation dose ≥8 μSv/h; that of 50–100 km marginally included areas with an air radiation dose ≥4 μSv/h at the time of the accident [3, 12, 13].

### Outflow of physicians

We defined outflow of physicians in terms of those who transferred between the pre- and post-disaster year to a municipality (city, town, or village) that was located farther from FDNPP (far-city outflow). We measured the distance from the centroid point of the municipality where the physician worked to the FDNPP site. In a supplementary analysis, we defined another type of outflow, which was that of transferring from the original prefecture (Fukushima or Miyagi) to another prefecture (prefecture outflow).

### Physician characteristics

We used data on five physician characteristics: type of facility (clinic or hospital) where they worked; clinical specialty; age; career length; and sex. We classified clinical specialty into five subcategories: internal medicine; surgery; psychiatry; other; and resident doctors. Information on age and years of experience was collected from the 2012 survey. We obtained other variables from the 2010 survey. Since 2004, physicians in Japan have been required to undertake a 2-year postgraduate clinical training program [14, 15]. In this study, we use the term “resident” to signify a physician who was undergoing postgraduate clinical training. We classified age into two categories for the main analysis: ≤35 and >35years. The age of 35 usually means about 10 years since graduation. We calculated career length based on the date of registration in the survey and classified it into two categories: ≤20 and >20 years. This normally signifies an additional 20 years after graduation; however, age and years of experience were not perfectly concordant. For a more detailed analysis of physician characteristics, we classified age into four categories: ≤35, 36–50, 51–65, and ≥66 years. We divided career length into six categories: ≤2, 3–5, 6–10, 11–20, 21–30, and ≥31 years.

### Statistical analysis

We evaluated the outflow of physicians in two ways: (1) evaluation for the decrease or increase in the number of physicians with a certain characteristic between the pre- and post-disaster year; (2) evaluation for the association between geographic or individual characteristics and physician outflow. In the first evaluation, to obtain the overall trend, we determined the number of physicians and number of physicians per 100,000 population; we then calculated the change between the pre- (2010) and post- (2012) disaster year. We classified the results according to the location: prefecture and FDNPP-distance category in the case of Fukushima and Miyagi. Then, we stratified the number and proportional change according to physician characteristics. To compare the proportional change in affected areas with the control, we counted the number of physicians in Iwate in the eastern Tohoku region and all three prefectures of the western Tohoku region (Aomori, Akita, and Yamagata). To determine geographic distribution, we calculated the change in the total number of physicians in each city, and we then entered those details on a map. To calculate the proportional change in the number of physicians to the overall population, we obtained population data for each municipality from the Survey on the Basic Resident Registration, conducted by the Ministry of Internal Affairs and Communications in 2009 (pre-disaster) and 2012 (post-disaster).

In the second evaluation, we excluded physicians who worked within the area ≤20km from FDNPP, which was the restricted area. In addition, some physicians may have moved to other places as a direct result of the tsunami; thus, we removed from the analysis municipalities that were located within 5 km of the tsunami-affected coast. We obtained information about the tsunami-affected area from the Geospatial Information Authority of Japan, Ministry of Land, Infrastructure, Transport and Tourism [10]. The study areas for this analysis appear in Fig 1. In the statistical analysis, we first calculated crude and adjusted odds ratios (ORs) and 95% confidence intervals (CIs) to examine the association between the distance from FDNPP and outflow of physicians using a logistic regression model. We selected the farthest group (>100 km) from FDNPP as the reference category. We adjusted the physicians’ characteristics with respect to prefecture (Miyagi or Fukushima), facility (clinic or hospital), being a resident (yes or no), career length (≤20 years or above), and sex. In addition, we examined the association between each physician characteristic and outflow. A *P* value of <0.05 (two-sided test) was considered statistically significant.

We evaluated which types of physicians were more likely to leave the disaster area, taking into account the proximity to FDNPP. First, to determine the susceptibility to the effects of proximity to FDNPP with respect to physician outflow, we evaluated the statistical interaction between each category of physician characteristic and proximity to FDNPP by including an interaction term. We then calculated adjusted ORs for the associations between proximity to FDNPP and physician outflow, stratified according to each physician category using the logistic regression model. In this step, we included the same variables as in the fully adjusted model. Owing to the high correlation coefficient between age and career length (correlation coefficient = 0.51, *P* <0.01), we excluded career length and age (≤35 years or above) from the analysis stratified by age and career length, respectively.

In the supplementary analysis, we conducted the same analysis using the prefecture-level outflow of physicians. We performed statistical analysis in IBM SPSS Statistics, version 22 (IBM Japan Inc., Tokyo, Japan).

## Results

Table 1 shows the number and proportional change in the population and physicians in each prefecture and in each distance range from FDNPP for Miyagi and Fukushima. The population decreased over the study area, especially in Fukushima (–3.5%). It was particularly conspicuous in municipalities located ≤20 km (–7.2%) and 20–50km (–3.9%) from FDNPP. The population also showed a decline (–3.7%) in the area >100 km, which included the tsunami-affected area. The numbers in the comparison areas (Iwate and western Tohoku) appear in S1 Table. When we focused on Miyagi and Fukushima prefectures, the decrease in number of physicians was most conspicuous in Fukushima (–5.3%); however, the number increased in Miyagi (2.8%) and the reference area of Iwate (2.4%). The decrease in Fukushima and increase in Miyagi and Iwate were evident even after taking the prefecture’s population change into account (change in physician to population ratios: –1.9%, 3.2%, and 4.8%, respectively). The number of physicians also decreased with the decrease in distance from FDNPP. Municipalities located ≤20 km from FDNPP were within the restricted area; there was thus a marked decrease in the number of physicians in such municipalities (–23.2%). In the areas 20–50 km and 50–100 km from FDNPP, we also observed a decrease in the number of physicians (–6.3% and –2.2%, respectively); however, there was an increase in the area >100 km from FDNPP (19.5%). The decrease in the number of physicians in Fukushima and in the area within 100 km from FDNPP was evident even after taking the population decline into account. Notably, physicians aged ≤35 years decreased in areas ≤20 km (–66.7%), 20–50 km (–18.0%), and 50–100 km (–14.8%) from FDNPP; they markedly increased in the area >100 km from FDNPP (64.2%).

**Table 1:** Number of physicians and proportional change according to prefecture and distance from the Fukushima Daiichi Nuclear Power Plant in the pre- and post-disaster years.

Sub-category	Tohoku-east sub region (Prefecture level)										Distance from FDNPP (km) in Miyagi and Fukushima																			
	Miyagi					Fukushima					≤20.0					20–50					50–100					>100				
	Pre, N	(%)	Post, N	(%)	P/C	Pre, N	(%)	Post, N	(%)	P/C	Pre, N	(%)	Post, N	(%)	P/C	Pre, N	(%)	Post, N	(%)	P/C	Pre, N	(%)	Post, N	(%)	P/C	Pre, N	(%)	Post, N	(%)	P/C
**Population**^†^	2329		2319		-0.5	2052		1980		-3.5	188		175		-7.2	518		497		-3.9	2822		2806		-0.6	853		821		-3.7
**Physicians**	4938		5075		2.8	3703		3506		-5.3	177		136		-23.2	1693		1586		-6.3	5689		5566		-2.2	1082		1293		19.5
**Physician to population ratios**^‡^	212		219		3.2	180		177		-1.9	94		78		-17.2	327		319		-2.5	202		198		-1.6	127		157		24.1
**Type of facility**																														
Clinic	1675	(34)	1750	(34)	4.5	1420	(38)	1338	(38)	-5.8	78	(44)	50	(37)	-35.9	712	(42)	700	(44)	-1.7	1865	(33)	1866	(34)	0.1	440	(41)	472	(37)	7.3
Hospital	3263	(66)	3325	(66)	1.9	2283	(62)	2168	(62)	-5.0	99	(56)	86	(63)	-13.1	981	(58)	886	(56)	-9.7	3824	(67)	3700	(66)	-3.2	642	(59)	821	(63)	27.9
**Clinical specialty**																														
Internal Medicine	2130	(43)	2217	(44)	4.1	1663	(45)	1567	(45)	-5.8	83	(47)	66	(49)	-20.5	768	(45)	713	(45)	-7.2	2423	(43)	2420	(43)	-0.1	519	(48)	585	(45)	12.7
Surgery	1133	(23)	1170	(23)	3.3	889	(24)	869	(25)	-2.2	41	(23)	30	(22)	-26.8	411	(24)	393	(25)	-4.4	1322	(23)	1294	(23)	-2.1	248	(23)	322	(25)	29.8
Psychiatry	289	(6)	281	(6)	-2.8	222	(6)	196	(6)	-11.7	23	(13)	17	(13)	-26.1	92	(5)	82	(5)	-10.9	336	(6)	308	(6)	-8.3	60	(6)	70	(5)	16.7
Other	1155	(23)	1196	(24)	3.5	782	(21)	751	(21)	-4.0	30	(17)	23	(17)	-23.3	356	(21)	335	(21)	-5.9	1364	(24)	1341	(24)	-1.7	187	(17)	248	(19)	32.6
Resident	231	(5)	211	(4)	-8.7	147	(4)	123	(4)	-16.3	0	(0)	0	(0)	0	66	(4)	63	(4)	-4.5	244	(4)	203	(4)	-16.8	68	(6)	68	(5)	0.0
**Age**																														
≤35 years	1083	(22)	1059	(21)	-2.2	683	(18)	576	(16)	-15.7	18	(10)	6	(4)	-66.7	267	(16)	219	(14)	-18.0	1294	(23)	1103	(20)	-14.8	187	(17)	307	(24)	64.2
36–50	1813	(37)	1821	(36)	0.4	1284	(35)	1135	(32)	-11.6	63	(36)	49	(36)	-22.2	575	(34)	488	(31)	-15.1	2101	(37)	2014	(36)	-4.1	358	(33)	405	(31)	13.1
51–65	1411	(29)	1561	(31)	10.6	1172	(32)	1233	(35)	5.2	62	(35)	56	(41)	-9.7	567	(33)	596	(38)	5.1	1580	(28)	1731	(31)	9.6	374	(35)	411	(32)	9.9
≥66	631	(13)	634	(12)	0.5	564	(15)	562	(16)	-0.4	34	(19)	25	(18)	-26.5	284	(17)	283	(18)	-0.4	714	(13)	718	(13)	0.6	163	(15)	170	(13)	4.3
**Career length**																														
≤2 years	218	(4)	197	(4)	-9.6	151	(4)	126	(4)	-16.6	0	(0)	0	(0)	0	65	(4)	63	(4)	-3.1	250	(4)	199	(4)	-20.4	54	(5)	61	(5)	13.0
3–5	347	(7)	348	(7)	0.3	231	(6)	197	(6)	-14.7	8	(5)	0	(0)	-100.0	98	(6)	68	(4)	-30.6	406	(7)	356	(6)	-12.3	66	(6)	121	(9)	83.3
6–10	673	(14)	698	(14)	3.7	427	(12)	365	(10)	-14.5	16	(9)	13	(10)	-18.8	149	(9)	126	(8)	-15.4	836	(15)	752	(14)	-10.0	99	(9)	172	(13)	73.7
11–20	1241	(25)	1204	(24)	-3.0	831	(22)	702	(20)	-15.5	45	(25)	29	(21)	-35.6	369	(22)	290	(18)	-21.4	1417	(25)	1323	(24)	-6.6	241	(22)	264	(20)	9.5
≥21	2459	(50)	2628	(52)	6.9	2063	(56)	2116	(60)	2.6	108	(61)	94	(69)	-13.0	1012	(60)	1039	(66)	2.7	2780	(49)	2936	(53)	5.6	622	(57)	675	(52)	8.5
**Sex**																														
Men	4144	(84)	4205	(83)	1.5	3156	(85)	3015	(86)	-4.5	164	(93)	127	(93)	-22.6	1449	(86)	1361	(86)	-6.1	4704	(83)	4589	(82)	-2.4	983	(91)	1143	(88)	16.3
Women	794	(16)	870	(17)	9.6	547	(15)	491	(14)	-10.2	13	(7)	9	(7)	-30.8	244	(14)	225	(14)	-7.8	985	(17)	977	(18)	-0.8	99	(9)	150	(12)	51.5

P/C, proportional change; Pre, pre-disaster (2010); Post, post-disaster (2012)

^†^ Thousand people.

^‡^ Physicians per 100,000 population.

The decreased number of physicians was observed in all clinical specialties within 100 km from FDNPP; it was particularly evident with psychiatrists: 20–50 km (–10.9%); and 50–100 km (–8.3%). In addition, there was an approximately 30% decrease in the number of physicians with a career length of 3–5 years in the area 20–50 km from FDNPP; however, such physicians showed an increase in areas >100 km (83%). The proportion of older physicians (>50 years) in 2010 (pre-disaster year) was higher in the area close to FDNPP than those most distant areas (≤20 km, 59%; 20–50 km, 56%; and >100 km, 45%); there was an increase in the proportions in closer areas in the post-disaster year of 2012 (≤20 km, 6% increase; 20–50 km, 5% increase; and >100 km, 5% decrease). These result suggest a more rapid aging of the physician population in areas close to FDNPP than in other areas.

**Fig 2:**
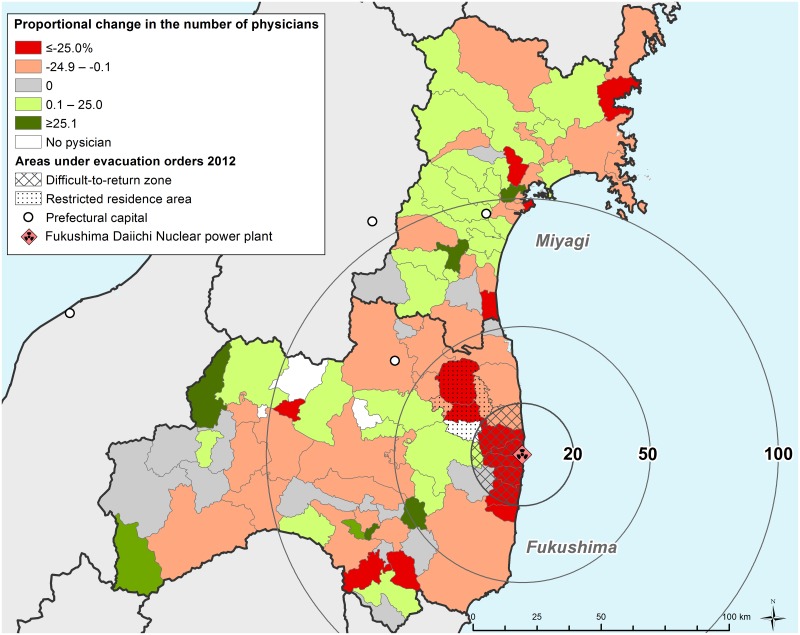
Proportional change in the number of physicians between the pre- and post-disaster years.

The geographic distribution of the proportional change in the number of physicians appears in Fig 2. The decrease in physician numbers was remarkable in the area around FDNPP in Fukushima Prefecture compared with Pacific coastal areas hit by the tsunami.

Table 2 shows the crude and adjusted association of the decrease in distance from FDNPP and physician characteristics with outflow from FDNPP of physicians who lived in Miyagi and Fukushima prefectures. Compared with physicians who lived >100 km from FDNPP, those who lived 20–50 km and 50–100 km from FDNPP showed an almost 4-fold and 3-fold, respectively, greater likelihood to leave their areas. The effect of proximity to FDNPP on physician outflow was still evident after adjustment for physician characteristics. In a separate evaluation according to physician characteristics, the increased outflow was particularly conspicuous among physicians who worked in hospitals, were younger (≤35 years), and had shorter career lengths (≤20 years).

**Table 2:** Crude and adjusted association of proximity to Fukushima Daiichi Nuclear Power Plant and physician characteristics with outflow from the area (N = 6,055).

	Total, N	(case, %^†^)	Single model		Full adjusted model^§^	
			OR	(95% CI)	OR	(95% CI)
**Distance from FDNPP**						
>100 km	614	(4.7)	1 (ref)		1 (ref)	
50–100 km	3844	(11.3)	2.6	(1.7–3.8)	2.0	(1.3–3.0)
20–50 km	1597	(16.0)	3.9	(2.6–5.7)	4.0	(2.5–6.4)
**Prefecture**						
Miyagi	2723	(9.5)	1(ref)			
Fukushima	3332	(13.8)	1.5	(1.3–1.8)		
**Type of facility**						
Clinic	2110	(2.2)	1(ref)			
Hospital	3945	(17.1)	9.2	(6.8–12.5)		
**Residents**						
No	5802	(10.5)	1(ref)			
Yes	253	(42.7)	6.3	(4.9–8.2)		
**Age**^‡^						
>35 years	4775	(5.9)	1(ref)			
≤35 years	1280	(34.4)	8.4	(7.1–9.9)		
**Career length**^‡^						
>20 years	3414	(2.9)	1(ref)			
≤20 years	2641	(23.6)	10.4	(8.4–13.0)		
**Sex**						
Men	5116	(11.5)	1(ref)			
Women	939	(14.2)	1.3	(1.0–1.6)		

CI, confidence interval; FDNPP, Fukushima Daiichi Nuclear Power Plant; OR, odds ratio; Ref, reference.

^†^ Proportion of cases in each distance group stratified by physician characteristics.

^‡^ Age and career length were at the post-disaster survey.

^§^ The fully adjusted model includes distance from FDNPP, prefecture, type of facility, resident status, career length, and sex.

**Fig 3:**
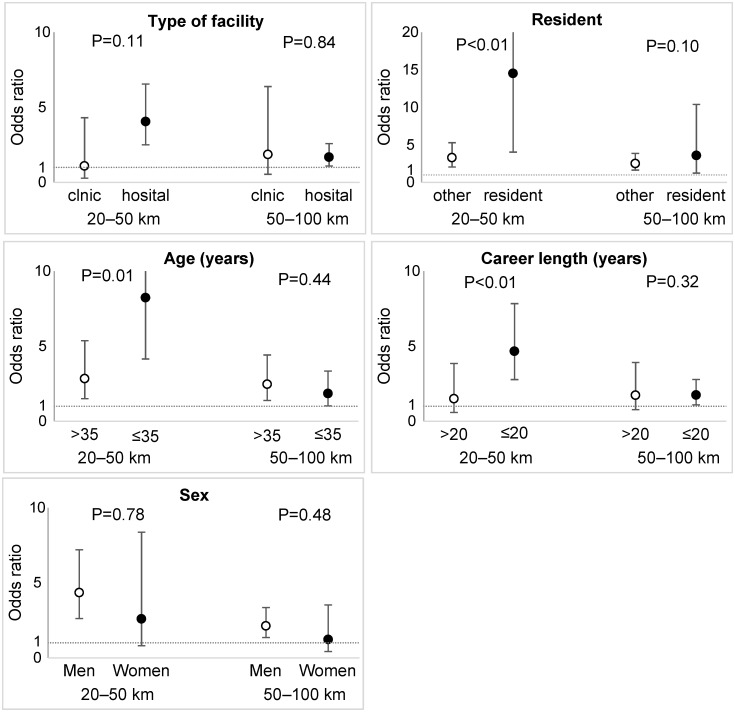
Association between proximity to Fukushima Daiichi Nuclear Power Plant and physician outflow stratified by physician characteristics (N = 6,055). The reference is the subgroup located >100 km from FDNPP. *P* for the interaction between distance to FDNPP and each characteristic. FDNPP, Fukushima Daiichi Nuclear Power Plant.

Fig 3 shows the ORs for the association between the distance from FDNPP and physician outflow stratified by physician characteristics (the reference subgroup is the area >100 km from FDNPP). The ORs in the distance of 20–50 km from FDNPP were higher among physicians who were residents (*P* for interaction <0.01), aged ≤35 years (*P* = 0.02), and had a shorter career length of ≤20 years (*P* <0.01) than other physicians. This mean that the effect of decreased distance to the disaster site on the physician outflow differed according to physician characteristics. The interaction terms for type of facility and sex were not statistically significant.

In the supplementary analysis, we examined the association between proximity to FDNPP and physician outflow to other prefectures. The results did not substantially change from those in the main analysis dealing with outflow to more distant municipalities (S2 Table and S1 Fig).

## Discussion

This study determined the character of the physician outflow from FDNPP after the Great East Japan Earthquake by comparing the pre- and post-disaster situations. In areas closer to FDNPP, younger physicians and those with shorter careers were more likely to leave than older physicians and those with longer careers. In addition, residents undergoing clinical training were more likely to depart for more distant areas. The outflow of physicians from FDNPP was more pronounced with decreasing distance from the plant.

One strength of this study is that we made a detailed evaluation of the role of physician characteristics in the post-disaster migration. Our findings are consistent with those of a previous study, which reported that female and younger practitioners were more likely to depart from areas badly affected by Hurricane Katrina in the United States in 2005 [16]. Even though the type of the disasters was different, such physicians may take similar actions following major disasters in future. In addition, after the nuclear accident, we observed that such physicians—except for females—were also sensitive to the distance from the disaster site.

The decline we observed in the number of physicians was most remarkable in the area close to FDNPP. In general, younger physicians are more likely to change their practice locations than older ones [17]; that was confirmed by our results. However, our findings further revealed that the outflow of young physicians outweighed their inflow to the FDNPP areas. Accordingly, it is an important question for future research as to whether these highly mobile young physicians later returned to areas close to FDNPP.

Health-care infrastructure factors, such as being unable to accept residents owing to malfunctioning hospitals, could affect the migration of younger physicians. Other feasible reasons for outflow of younger physicians and those earlier in their careers could be that they had small children and were sensitive to the children’s expose to radiation, as has been reported in the media [18]. Except for the evacuation zone, which contained 12 municipalities [19], the monthly average air radiation dose rates in surrounding areas were less than 0.025 μSv/h; a few municipalities had rates of 0.025–0.05 μSv/h at the time of the post-disaster survey in December 2012 [20]. However, the majority of people in the area, especially younger ones, still had a fear of radiation exposure [21, 22]. The period of 3–10 years after graduation from medical school is critically important for physicians in deciding on their future place of work [17]. Even before the disaster, the study areas were rural and medically underserved [23, 24]. That existing shortage of physicians was exacerbated by the post-disaster outflow of younger physicians.

One study from Japan reported that the total number of nurses also decreased in some areas following the Great East Japan Earthquake [25]. It is notable that the number of physicians per 100,000 population likewise decreased in the area close to FDNPP; that decline was almost the same as the reduction in the number of nurses [25]. Thus, the impact of the nuclear accident may be similar among different health-care professionals, although we observed a more evident effect on young physicians in this study.

A major limitation of this study is that data availability demanded that we use data on physician numbers 21 months after the Great East Japan Earthquake occurred. Some physicians temporarily working in disaster areas as part of post-disaster aid programs could have been included in our dataset. Despite the possibility of such overestimation, the decreased number of physicians was still evident around FDNPP. However, we were unable to count the number of physicians who were dispatched from hospitals outside the disaster area for reconstruction assistance. Although the presence of such physicians may have led to an underestimation of the number of physicians in the post-disaster period, most of those physicians returned to their original facilities within several months. Thus, such an underestimation would be marginal by the end of 2012, when we obtained our post-disaster data. For example, the Japan Medical Association dispatched teams to disaster areas of the Great East Japan Earthquake [26]. The main activity of the dispatched medical teams terminated 4 months after the earthquake.

The severe shortage of younger physicians around FDNPP after the disaster implies that the actual work force decreased more than the decrease in number of physicians. In the area around FDNPP, the age structure of the population may have drastically changed, and it became older than in other areas [27]. That raised the medical care needs for the population as a whole. In addition, in the case of nuclear accidents, people suffer a long-term psychological burden [28]. The shortage of physicians found in the present study would lead to worsening health care in the affected areas. An intensive policy to support younger and early-career physicians should be instigated by local and national governments.

## Supporting Information

S1 Fig Association between proximity to Fukushima Daiichi Nuclear Power Plant and physician outflow from the prefecture stratified by physician characteristics (N = 6,055). (PDF)

S1 Table Number and proportional change in physicians in the comparison area in the pre- and post-disaster years. (PDF)

S2 Table Crude and adjusted association of proximity to Fukushima Daiichi Nuclear Power Plant and other physician characteristics with their outflow from the prefecture (N = 6,055). (PDF)
