# Supplementary material for: Characteristics of Physician Outflow from Disaster Areas following the Great East Japan Earthquake
Source: PLoS One. 2017 Jan 3;12(1):e0169220. doi: 10.1371/journal.pone.0169220 (PMC5207640; doi:10.1371/journal.pone.0169220)
Supplement: S1 Fig — (PDF) [file pone.0169220.s001.pdf]

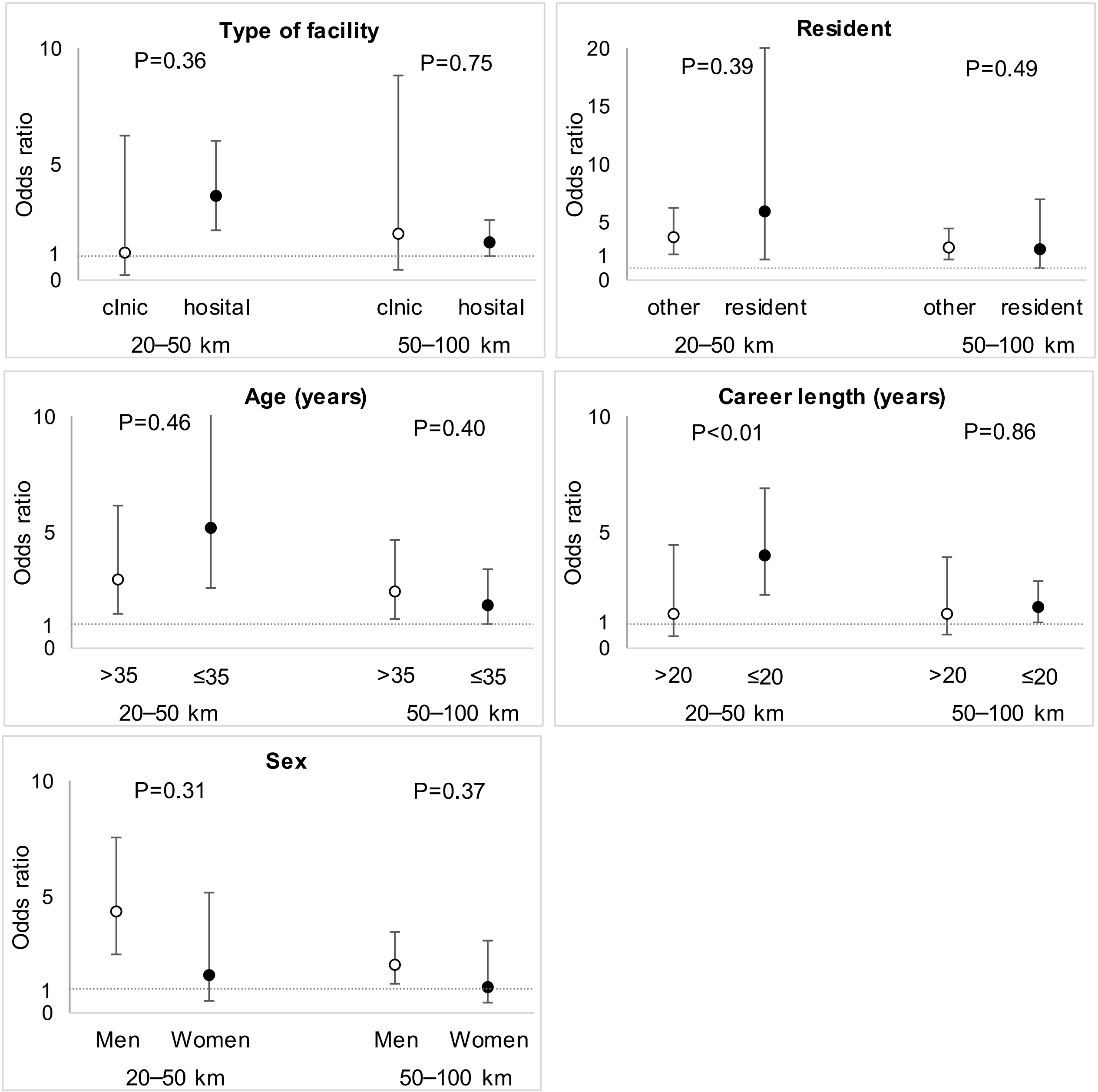

Fig S1. Association between proximity to Fukushima Daiichi Nuclear Power Plant and physician outflow from the prefecture stratified by physician characteristics (N=6,055)

The reference is the subgroup located >100 km from FDNPP. *P* for interaction between distance to FDNPP and each characteristic. FDNPP, Fukushima Daiichi Nuclear Power Plant.
