# Supplementary material for: Characteristics of Physician Outflow from Disaster Areas following the Great East Japan Earthquake
Source: PLoS One. 2017 Jan 3;12(1):e0169220. doi: 10.1371/journal.pone.0169220 (PMC5207640; doi:10.1371/journal.pone.0169220)
Supplement: S1 Table — (PDF) [file pone.0169220.s002.pdf]

Table S1. Number and proportional change in physicians in the comparison area in the pre- and post-disaster years

	Tohoku-east sub-region (Iwate)					Tohoku-west sub-region (Aomori, Akita, Yamagata)				
**Sub-category**	Pre, N	(%)	Post, N	(%)	Proportional change	Pre, N	(%)	Post, N	(%)	Proportional change
**Population**†	1345		1314		-2.3	3691		3604		-2.3
**Physicians**	2413		2471		2.4	7129		7116		-0.2
**Physician to population proportion**‡	179		188		4.8	193		197		2.2
**Type of facility**										
Clinic	850	(35)	847	(34)	-0.4	2465	(35)	2406	(34)	-2.4
Hospital	1563	(65)	1624	(66)	3.9	4664	(65)	4710	(66)	1.0
**Clinical specialty**										
Internal Medicine	1028	(43)	1058	(43)	2.9	3007	(42)	2995	(42)	-0.4
Surgery	611	(25)	614	(25)	0.5	1735	(24)	1738	(24)	0.2
Psychiatry	114	(5)	131	(5)	14.9	435	(6)	437	(6)	0.5
Other	520	(22)	534	(22)	2.7	1538	(22)	1560	(22)	1.4
Resident	140	(6)	134	(5)	-4.3	414	(6)	386	(5)	-6.8
**Age**										
≤35 years	482	(20)	511	(21)	6.0	1490	(21)	1469	(21)	-1.4
36–50	816	(34)	783	(32)	-4.0	2491	(35)	2374	(33)	-4.7
51–65	752	(31)	816	(33)	8.5	2158	(30)	2318	(33)	7.4
≥66	363	(15)	361	(15)	-0.6	990	(14)	955	(13)	-3.5
**Career length**										
≤2 years	142	(6)	133	(5)	-6.3	394	(6)	373	(5)	-5.3
3–5	162	(7)	175	(7)	8.0	476	(7)	498	(7)	4.6
6–10	262	(11)	288	(12)	9.9	856	(12)	835	(12)	-2.5
11–20	535	(22)	508	(21)	-5.0	1686	(24)	1542	(22)	-8.5
≥21	1312	(54)	1367	(55)	4.2	3717	(52)	3868	(54)	4.1
**Sex**										
Men	2067	(86)	2100	(85)	1.6	6064	(85)	5985	(84)	-1.3
Women	346	(14)	371	(15)	7.2	1065	(15)	1131	(16)	6.2

Pre, pre-disaster (2010); Post, post-disaster (2012)

† Thousand people.

‡ Physicians per 100,000 population.
