# Supplementary material for: Characteristics of Physician Outflow from Disaster Areas following the Great East Japan Earthquake
Source: PLoS One. 2017 Jan 3;12(1):e0169220. doi: 10.1371/journal.pone.0169220 (PMC5207640; doi:10.1371/journal.pone.0169220)
Supplement: S2 Table — (PDF) [file pone.0169220.s003.pdf]

Table S2. Crude and adjusted association of proximity to Fukushima Daiichi Nuclear Power Plant and other physician characteristics with their outflow from the prefecture (N=6,055)

	Total, N	(case, %†)	Single model		Full adjusted model§	
			**OR**	**(95% CI)**	**OR**	**(95% CI)**
**Distance from FDNPP**						
>100 km	614	(3.9)	1(ref)		1(ref)	
50–100 km	3844	(9.1)	2.5	(1.6-3.7)	1.9	(1.2-2.9)
20–50 km	1597	(12.0)	3.3	(2.2-5.2)	3.6	(2.2-6.0)
**Prefecture**						
Miyagi	2723	(8.0)	1(ref)			
Fukushima	3332	(10.4)	1.3	(1.1-1.6)		
**Type of facility**						
Clinic	2110	(1.5)	1(ref)			
Hospital	3945	(13.5)	10.1	(7.1-14.5)		
**Residents**						
No	5796	(8.3)	1(ref)			
Yes	253	(33.2)	5.5	(4.2-7.3)		
**Age‡**						
>35 years	4775	(4.4)	1(ref)			
≤35 years	1280	(27.8)	8.5	(7.0-10.2)		
**Career length‡**						
>20 years	3414	(2.0)	1(ref)			
≤20 years	2641	(18.8)	11.6	(8.9-15.0)		
**Sex**						
Men	5116	(8.9)	1(ref)			
Women	939	(11.8)	1.4	(1.1-1.7)		

CI, confidence interval; FDNPP, Fukushima Daiichi Nuclear Power Plant; OR, odds ratio; Ref, reference.

† Proportion of cases in each distance group stratified by physician characteristics.

‡ Age and career length were at the post-disaster survey.

§ Fully adjusted models include distance from FDNPP, prefecture, type of facility, residents, career length, and sex.
